# Society Meetings

**Published:** 1909-01

**Authors:** 


### SOCIETY MEETINGS.

The Medical Society of the State of New York will hold its 103d annual meeting at Albany, January 25-27, 1909, under the presidency of Dr. Arthur G. Root, of Albany.

The Medical Society of the County of Erie held its 87th annual meeting December 21, 1908, under the presidency of Dr. Edward Clark. An interesting program was disposed of.

The Buffalo Academy of Medicine held meetings during the month of December as follows:

*Section on Surgery.*—Regular meeting held Tuesday evening, December 1, 1908. Program: Symposium on fractures. (1) Use of the *x*-ray in fractures, W. W. Plummer; (2) The non-operative treatment of fractures, Frank J. Carr; (3) The operative treatment of fractures, Marshall Clinton; (4) Complications in treatment of fractures, Roswell Park. Discussion opened by Eugene A. Smith and Edgar R. McGuire.

*Section on Medicine.*—Regular meeting was held Tuesday evening, December 8, 1908. Program: A study of the effects of tedious labors and forceps deliveries in the production of epilepsy, spastic paralysis and idiocy, James W. Putnam. Discussion was opened by Dr P. W. Van Peyma from the obstetric standpoint and by Dr. Irving M. Snow from the pediatric standpoint.

*Section of Obstetrics and Gynecology.*—Regular meeting was held Tuesday evening, December 15, 1908. Program. (1) Manual dilatation of parturient canal, P. W. Van Peyma; (2) Education of women relative to diseases incident to menopause, De Witt G. Wilcox.

*Section on Pathology.*—A stated meeting of the Academy was held Tuesday evening, December 22, 1908. Program: Absorption from the peritoneal cavity, W. G. MacCallum, Associate Professor of Pathology and Curator of the Museum at Johns Hopkins University, Baltimore, Md. A meeting of the council was held at 8.15 p. m. A collation was served at the close of the meeting.

---

The Rochester Academy of Medicine held meetings during the month of December at the Hotel Seneca, as follows:

*Section on Surgery.*—Regular meeting was held Wednesday evening, December 9, 1908. Program: (1) Deviations and spurs of the nasal septum and their removal by submucous resection, Nathan D. MacDowell. (2) Operative lateral sinus thrombosis, Bradford A. Richards,.

*Section on Obstetrics.*—Regular meeting was held Wednesday evening, December 16, 1908. Program: Case reports of cesarean section William M. Brown. Discussion opened by Edward W. Mulligan.

